# The Impact of Severe Albuminuria on the Proinflammatory Monocyte Subset CD14++CD16+ and Antigen-Specific Immune Responses

**DOI:** 10.3390/ijms27177866

**Published:** 2026-09-02

**Authors:** Humberto Luna Sandoval, Christof Ulrich, Silke Markau, Matthias Girndt

**Affiliations:** 1 Department of Internal Medicine II, Martin Luther University Halle-Wittenberg, Ernst-Grube-Str. 29, 06120 Halle (Saale), Germany; humberto.lunasandoval@uk-halle.de (H.L.S.); christof.ulrich@uk-halle.de (C.U.); silke.markau@uk-halle.de (S.M.); 2 KfH Nierenzentrum Halle, Bachstelzenweg 4, 06120 Halle (Saale), Germany

**Keywords:** chronic kidney disease, proteinuria, albuminuria, monocyte subsets, CD86, CD28, CD137, adaptive immune response, CMV, SARS-CoV-2

## Abstract

Loss of renal function is associated with premature aging of the immune system; however, earlier studies on this topic enrolled patients with reduced GFR and did not distinguish between those with or without proteinuria. Clinical observations suggest that proteinuria alone might also impair immune defense. It is thus an open question whether patients with a urinary albumin-to-creatinine ratio > 300 mg/g (severe albuminuria) independent of their eGFR have an inflammatory risk profile and a disturbed adaptive immune response. This cross-sectional study included 25 patients with severe albuminuria (uACR > 300 mg/g; P-Group), 19 patients with an eGFR < 60 mL/min 1.73 m2 and a uACR < 300 mg/g (G-Group), and 30 patients with adequate kidney function (C-group). All three cohorts have a similar cardiovascular background with arterial hypertension and coronary artery disease. PBMCs from the patients were challenged with the superantigen Staphylococcal enterotoxin B (SEB) as well as with CMV- and SARS-CoV-2-specific antigens. Monocyte subsets and CD86 and HLA-DR expression were determined through flow cytometry. Transcripts of CD28 and IFN-α and -γ were measured by qPCR. Compared to those in the C-group, patients in the G-group showed a higher polyclonal SEB response and had significantly elevated circulating numbers of inflammatory monocytes (subset CD14++CD16+). CD28 transcripts were decreased in the P-group and G-group compared to the C-group, reaching significance for the G-Group. The CMV- and SARS-CoV-2-specific immune response, as measured by the frequency of CD4+69+137+ T and CD8+69+137+ T cells, was comparable in all three groups. Severe albuminuria per se does not alter the antigen-specific immune response; however, patients with reduced GFR, but not those with proteinuria, show inflammatory and polyclonal immune activation.

## 1. Introduction

Chronic kidney disease (CKD) is a growing problem affecting about 10% of the general population worldwide [1,2]. Aside from typical risk factors such as hypertension, diabetes, and cardiovascular disease, infections are a major concern in CKD. Indeed, the condition itself increases the risk of developing severe acute and chronic infections, e.g., COVID-19 [3]. Among CKD patients, infections act as drivers of morbidity and mortality and contribute to higher hospitalization rates [4]. Impairment of the immune system in CKD leads to a higher rate of viral infections such as hepatitis, CMV, and COVID [5,6,7,8]. Failures to adequately respond to infection are due, at least in part, to impaired B7-2 (CD86) signaling, low lymphocyte numbers, and reduced expression of co-stimulatory CD28, hampering antigen recognition and antigen presentation [9,10]. These antigen-specific defects occur in the presence of enhanced inflammation. A further consequence of immune dysfunction is a low immune response to vaccination [5].

The classification of CKD relies on the determination of the estimated glomerular filtration rate (eGFR) and on albuminuria, estimated as albumin-to-creatinine ratio (uACR). Four stages of eGFR (ml/min/1.73 m^2^) classify the severity of chronic kidney disease (CKD): stage G2: 60–89; stage 3a: 45–59; stage 3b: 30–44; stage G4: 15–29 mL/min/1.73 m^2^. ACR categories comprise A1 (mild; <30 mg/g), A2 (moderate; 30–299 mg/g), and A3 (severe; ≥300 mg/g). Albuminuria is therefore a marker of glomerular damage, and recent studies demonstrate that even a slight increase in urinary albumin is linked to cardiovascular diseases in the general population [11]. Furthermore, several studies have found evidence that albuminuria is linked to inflammation [12,13]. This raises the question whether albuminuria has a significant impact not only on inflammation but also on specific immune response. Regarding inflammation, we focused on CD14++CD16+, a proinflammatory monocyte subset linked to cardiovascular disease [14]. To analyze immune response, we challenged isolated PBMCs of patients with SEB as a polyclonal stimulus with SARS-CoV-2- and CMV-specific antigens. These experiments are intended to provide insight into the cellular immune responsiveness of patients.

CD137, also known as TNFRSF9, is an important costimulatory molecule for T cells; its binding to its ligand CD137L is associated with the survival and proliferation of CD8+ T cells [15]. Recently, Wölfl and colleagues demonstrated that CD137 is a more uniform marker of antigen-specific activation than the production of a single cytokine. They showed that CD137 expression allows for detection of the full repertoire of CD8+ T cells after stimulation with CMVpp65 [16]. Later, Altosole et al. showed the method is viable for detection of SARS-CoV-2-specific stimulation [17]. A different method of analyzing antigen-specific responses was suggested by Schwarz et al. [18], who showed that the mRNA expression of CXCL10 is an appropriate means of assessing 0SARS-CoV-2-specific immune responses. CXCL10 mRNA expression appeared to correlate well with the intracellular IFN-γ response. The association seemed convincing as the chemokine CXCL10 is also known as an interferon-γ-induced protein. CXCL10 binds to the CXCR3 receptor to induce chemotaxis, apoptosis, and cell growth [19]. CXCL10 is involved in various infectious diseases and appears to be protective in the severe acute respiratory syndrome [20].

This study intends to separate the influence of reduced GFR or proteinuria on cellular immune markers and, in order to achieve this, compares both to a control group with normal renal function.

## 2. Results

### 2.1. Demographic Data

In this cross-sectional analysis, we compared three groups of patients with different albuminuria and eGFR. While patients from the P- and G-groups had median eGFR values of 41.2 and 25.4 mL/min/1.73 m^2^, respectively, patients from the C-group had normal kidney function with a median of 86.0 mL/min/1.73 m^2^ (Table 1). The primary diseases of patients within the P- and G-groups were glomerulopathies, while the overwhelming majority of patients in the C-group were treated for cardiovascular and lipid disorders. Common to all three groups was a high prevalence of systemic arterial hypertension. The use of angiotensin-converting enzyme (ACE) inhibitor or angiotensin receptor blocker (ARB) medication and the cardiovascular risk profile (coronary artery disease, CAD; cerebrovascular disease, CVD) did not differ between the cohorts. Most interestingly, inflammatory markers like C-reactive protein (CRP) and interleukin-6 (IL-6) were slightly elevated above reference levels in all three groups. As expected, the use of sodium glucose co-transporter-2 (SGLT2-) inhibitors and diuretics was more frequent in the P- and G-groups than in the C-group (Table 1).

### 2.2. Hematology Parameters

Total leukocyte numbers were similar across the three study groups. Compared to the C-group, patients in the P-group had significantly increased granulocytes and reduced lymphocytes. Patients in the G-group showed the highest levels of monocytes without reaching statistical significance (Table 2).

#### Monocyte Characteristics and Subsets

**Figure 1:**
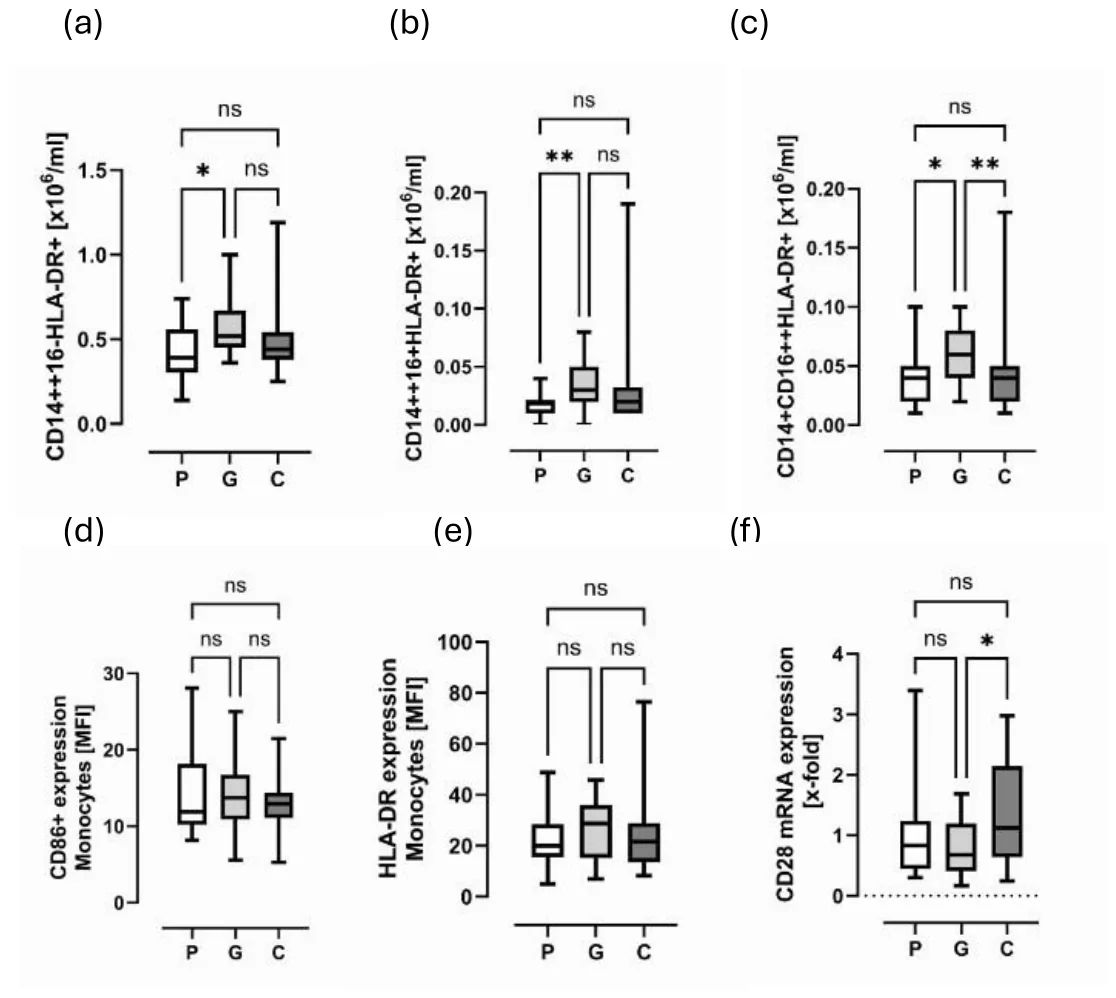
Absolute cell numbers of the 3 monocyte subsets (Mo1, Mo2, Mo3) and mean expression density (MFI) of HLA-DR as well as expression of the costimulatory molecules CD86 and CD28 mRNA in P-group, G-group and C-group. Mo1 cell number is depicted in (**a**), Mo2 in (**b**) and Mo3 in (**c**). (**d**) shows MFI of CD86-positive monocytes, (**e**) the MFI of HLA-DR-positive monocytes. CD28 mRNA expression in PBMCs of the 3 groups is presented in (**f**). Data is resented as box plot with the median, the 25/75 percentile, and the range and analyzed using one-way ANOVA with Tukey’s multiple comparison test as posttest or Kruskal-Wallis test as appropriate. * *p* < 0.05, ** *p* < 0.01, ns: not significant.

As shown in Table 2, patients in the G-group exhibited an expanded monocyte population compared to those in the P- and C-groups. The monocytes within the three patient groups were further subcategorized into different subsets (*classical Mo1*: CD14++CD16-; *intermediate Mo2*: CD14++CD16+; and *non-classical Mo3*: CD14+CD16++). As depicted in Figure 1, the G-group had the highest numbers of Mo1 (Figure 1a), Mo2 (Figure 1b), and Mo3 (Figure 1c).

Monocyte functionality can be characterized by the expression density (MFI in flow cytometry) of CD86, a major costimulatory molecule for T-cell activation, and HLA-DR, the MHC-class II molecule crucial to antigen presentation. The G-group showed significantly higher absolute monocytes (Table 2). Figure 1d illustrates the comparable median fluorescence intensity (MFI) of the co-stimulatory molecule CD86 in the three cohorts. Given the importance of HLA-DR for the presentation of antigenic peptides, we also measured the expression of this MHC-class II molecule on monocytes. However, there were again no significant differences in the MFI of HLA-DR on monocytes between the three cohorts (Figure 1e). Beyond CD86 (also called B7.2) expression on monocytes/antigen-presenting cells, detection of the expression density of CD28, the main ligand on T cells, also reflects the function of the CD28/B7 costimulatory pathway. As demonstrated in Figure 1f, there is a tendency for lower CD28 mRNA expression in patients with renal dysfunction compared to our control group. This effect reached statistical significance in the G-group (Figure 1f).

### 2.3. Lymphocyte Activation In Vitro

**Figure 2:**
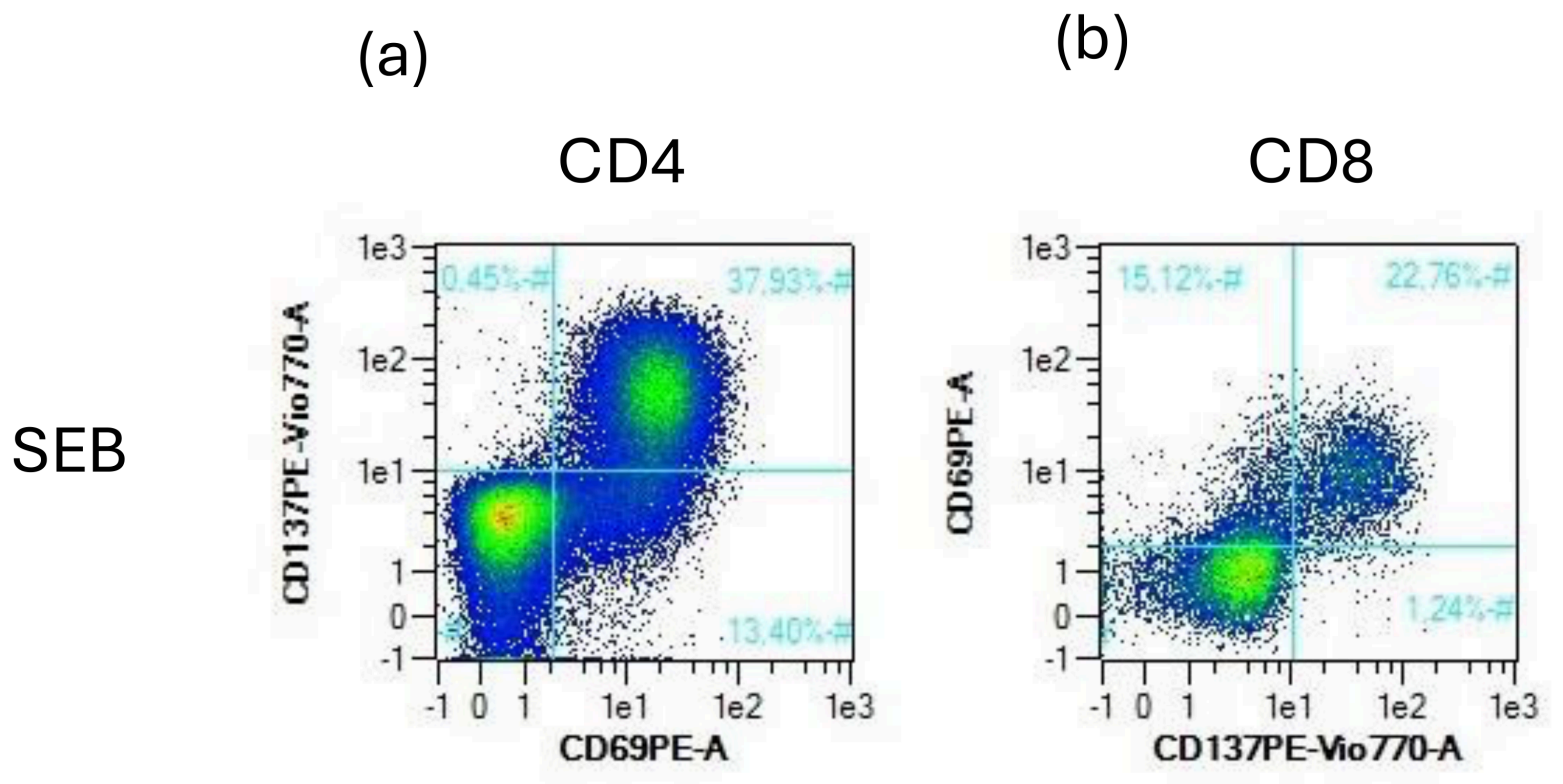
Representative dot plots showing the frequency of CD4+69+137+ (**a**) and CD8+69+137+ (**b**) after SEB stimulation (upper right segment in each of the dot plots) of a patient in G-group. Colors indicate event density, with blue representing lower and green/yellow/red representing progressively higher event densities.

To gain insight into the immune response capacity, we initially stimulated the PBMCs with staphylococcus enterotoxin B (SEB), a potent bacterial superantigen that induces a very strong T-cell response not restricted to individual antigen-specific clones. As reported, about 20% of all T cells can be activated by a high SEB concentration, which may be a proxy for a maximal immune response (Figure 2) [21]. We used the expression of CD69 and CD137 as activation markers that are useful for quantifying early lymphocyte responses.

**Figure 3:**
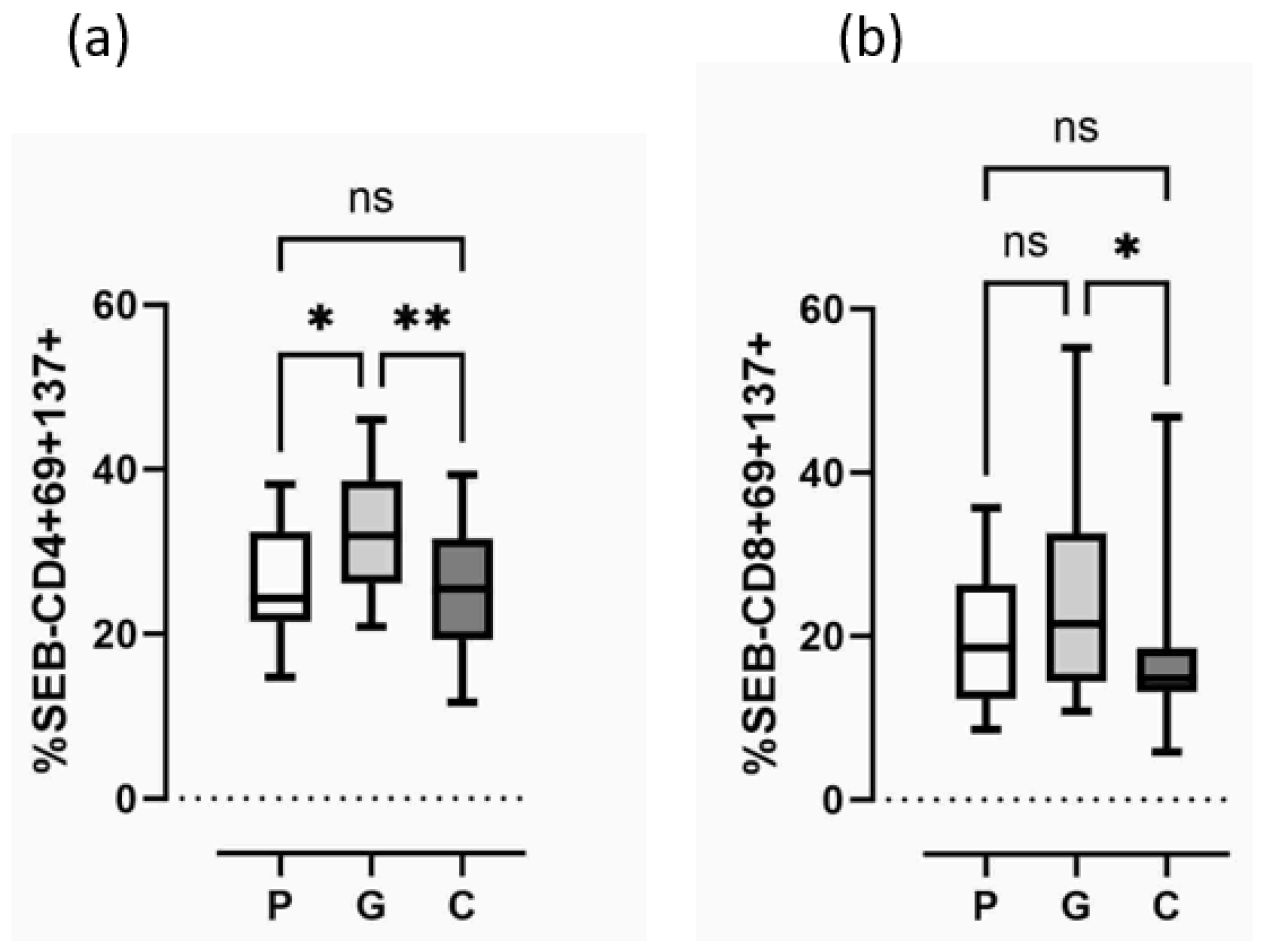
Polyclonal stimulation with staphylococcal enterotoxin B (SEB) in controls (C), G- and P- patients. (**a**) depicts the fraction of CD4+CD69+CD137+ cells among all CD4+, (**b**) the fraction of CD8+CD69+CD137+ cells after stimulation with SEB. Data are represented as box plot with the median and the 25/75 percentile and analyzed using one-way ANOVA with Tukey’s multiple comparison test as posttest. * *p* < 0.05, ** *p* < 0.01, ns: not significant.

As depicted in Figure 3, the stimulation of PBMCs with SEB resulted in a slightly higher increase in CD4+CD69+CD137+ and CD8+CD69+CD137+ in the G-group, as compared to the C-group (Figure 3a,b).

### 2.4. Specific Activation of T Cells with CMV- and SARS-CoV-2-Antigens

**Figure 4:**
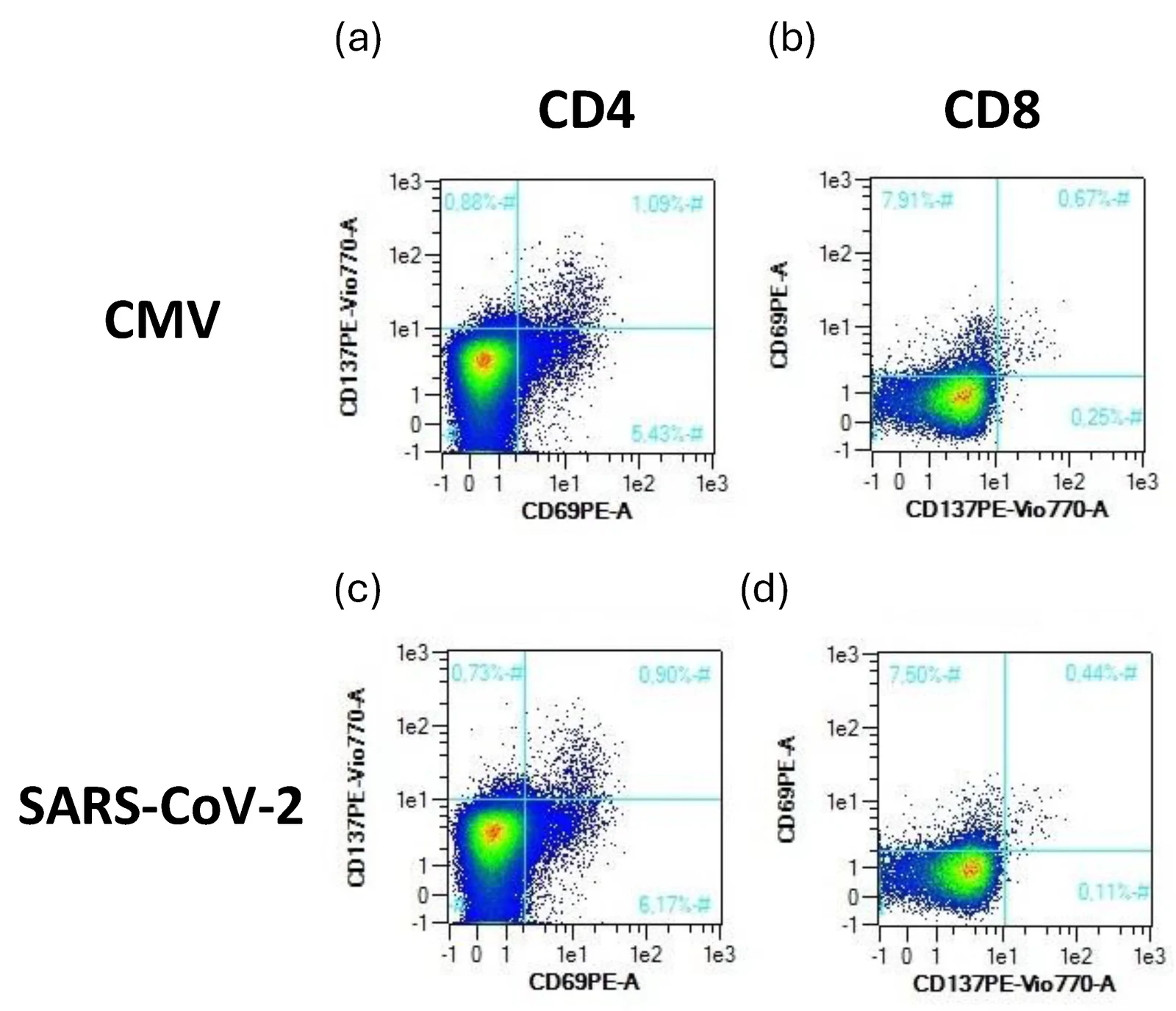
Representative density blots showing the frequencies of CMV-specific CD4+69+137+ (**a**) and CD8+69+137+ (**b**) as well as SARS-CoV-2-specific CD4+69+137+ (**c**) and CD8+69+137+ (**d**) T cells of a patient in G-group. Colors indicate event density, with blue representing lower and green/yellow/red representing progressively higher event densities.

To analyze the immune-specific T-cell response, the patient’s PBMCs were challenged with CMV- and SARS-CoV-2-specific antigens (see representative density blots in Figure 4).

**Figure 5:**
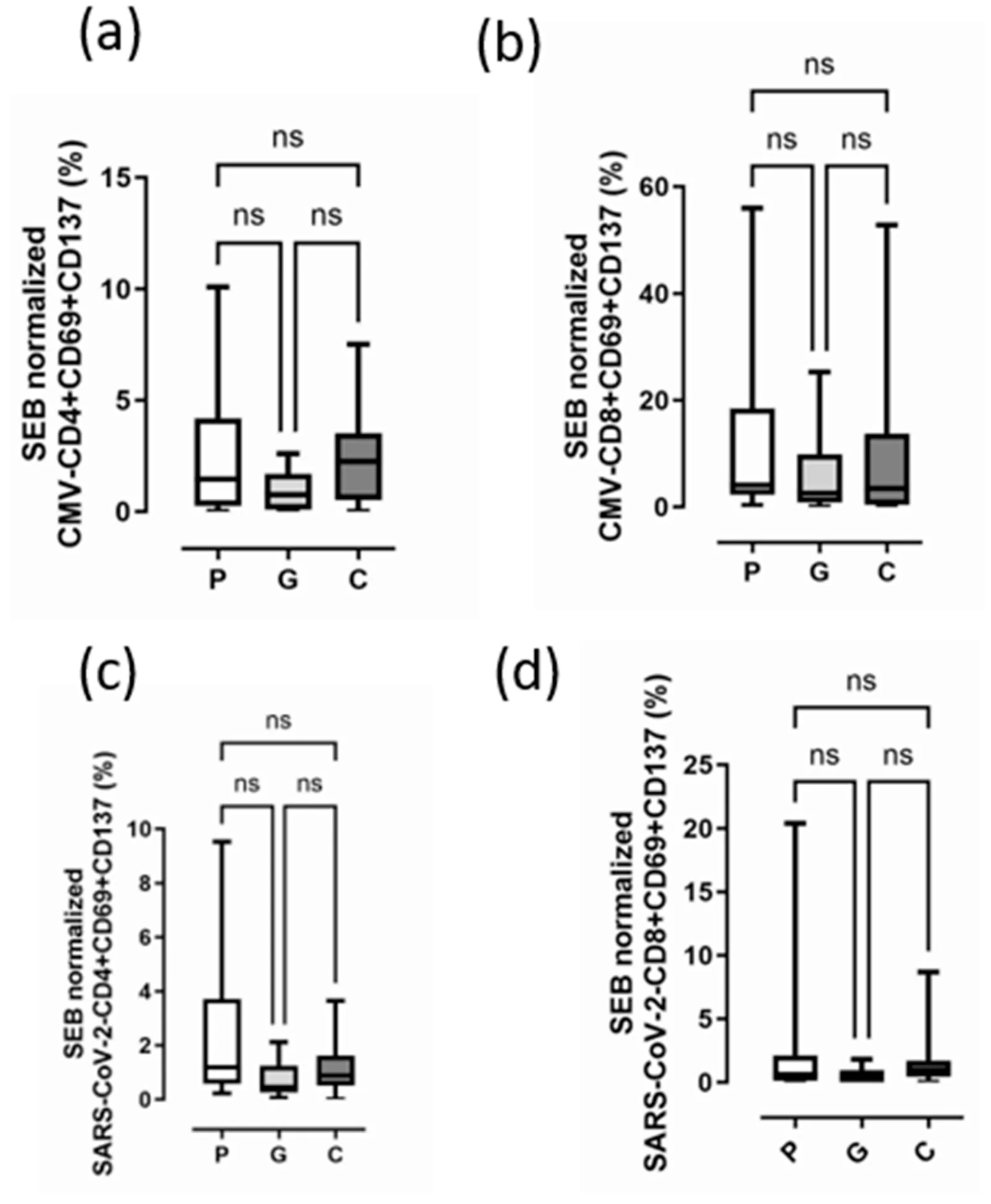
CMV- and SARS-CoV-2 specific stimulation after normalization to SEB stimulation in controls (C), G-patients and P-patients. The frequencies of CMV-specific CD4+CD69+CD137+ and CD8+CD69+CD137+ immune response are shown in (**a**) and (**b**), respectively. The frequencies of SARS-CoV-2 specific CD4+CD69+CD137+ and CD8+CD69+CD137+ (**c**,**d**). Data are resented as box plot with the median and the 25/75 percentile and analyzed using one-way ANOVA with Tukey’s multiple comparison test as posttest or Kruskal-Wallis test as appropriate. ns: not significant.

As can be seen in Table 3 and Figure 5, stimulation with the CMV antigen results in similar CD4 T-cell and CD8 T-cell responses in the three groups (Table 3). Examining the state of CMV seropositivity, we found a comparable CMV burden in the three groups (P: 69%, G: 59%, and C: 50%; *p* = 0.6395). Further, cellular response to SARS-CoV-2-specific stimulation did not differ between the patient groups (Table 3). In an exploratory analysis, we related the antigen-specific response to the broad response to the superantigen SEB; patients of the G-group tended to recruit a lower fraction of their CD4 cells to CMV (Figure 5a) and SARS-CoV-2 (Figure 5c). This aspect could not be proven statistically (ANOVA CMV-CD4: *p* = 0.08), which is likely due to the limited size of the patient cohort.

### 2.5. Expression of CXCL10, IFN-γ, and IFN-α

**Figure 6:**
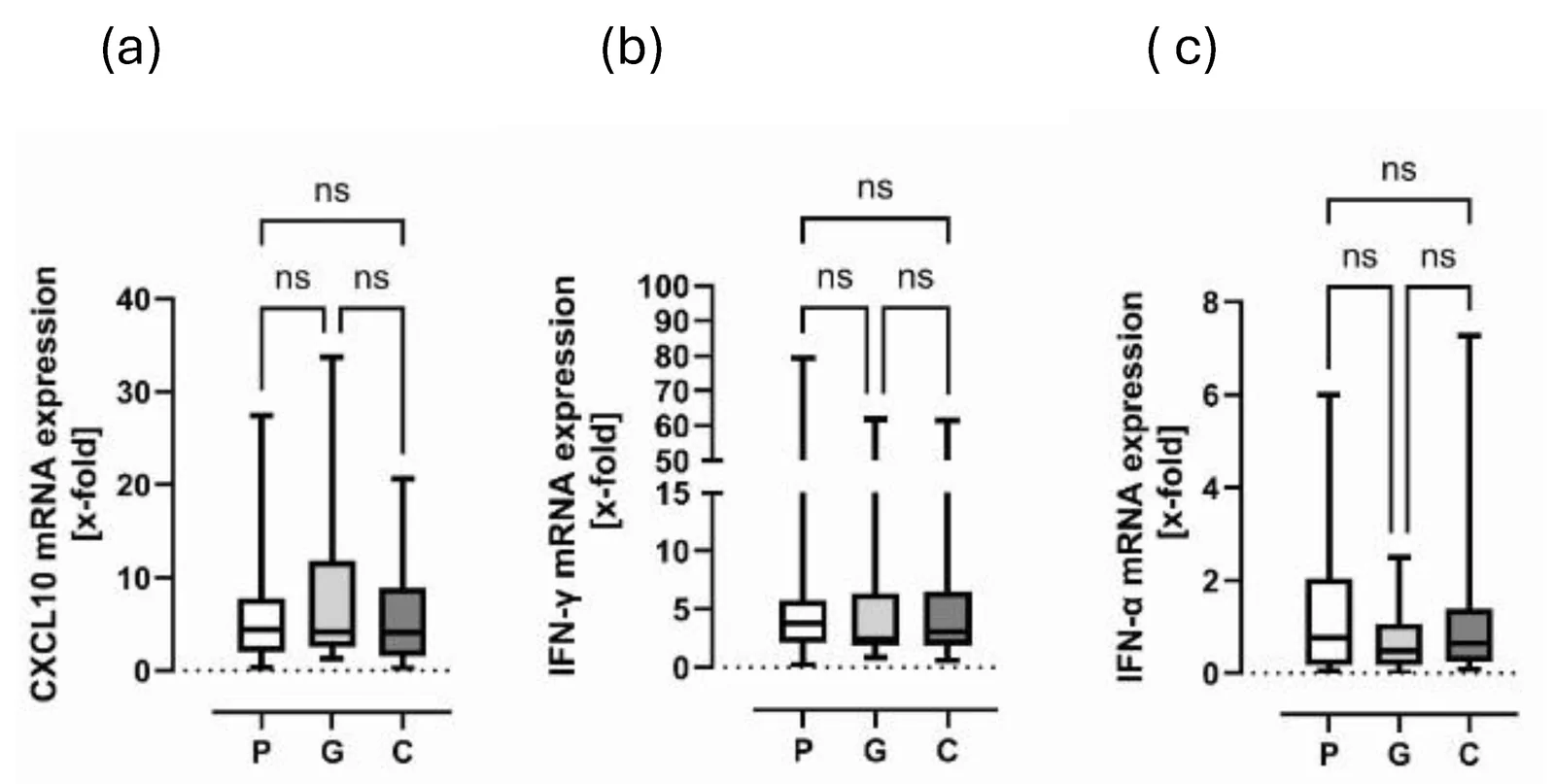
mRNA expression of CXCL10 and interferons in the 3 cohorts. (**a**) represents mRNA expression of CXCL10, (**b**) shows the mRNA expression of proinflammatory cytokine IFN-γ, (**c**) mRNA expression of the viral-specific IFN-α. Data is represented as box plot with the median and the 25/75 percentile or as single values and analyzed using one-way ANOVA with Tukey’s multiple comparison test or Kruskal-Wallis test as appropriate. as posttest. ns: not significant.

Chemokines and interferons are part of immune responses. To elucidate the role of CXCL10 in our study population, we measured mRNA expression of this chemokine. Figure 6a reveals that CXCL10 is not differently expressed in the three cohorts.

Additionally, in the context of virus-specific T-cell activation, mRNA expression of some interferon genes was analyzed. There was no significant difference in the expression of the inflammatory gene *IFN-γ* or the anti-viral gene *IFN-α* in the three groups (Figure 6b,c).

To determine systemic interferon levels, we measured IFN-γ and IFN-α in the serum of patients. As demonstrated in Table 3, there were no differences regarding either cytokine across the three groups (Table 4).

### 2.6. Correlation of CXCL10 Expression with Antigen-Specific Immune Responses

A correlation analysis was performed to evaluate the presumed relationship between CXCL10 and IFN-γ mRNA expression regarding CMV- and SARS-CoV-2-specific lymphocyte responses. While CMV-specific T-cell responses correlated significantly with IFN-γ mRNA expression, CXCL10 mRNA expression was associated with neither CMV- nor SARS-CoV-specific immune responses (Table 5).

## 3. Discussion

Chronic kidney disease results in multiple impairments in both the innate and adaptive immune systems [22]. The role of proteinuria as a causal factor has not been studied thoroughly, although clinical data suggest elevated infection risk in proteinuric patients with normal eGFR [23]. We show here that CKD-associated alterations in monocyte and monocyte subpopulation numbers, as well as lymphocyte activation, relate to impaired eGFR but not to proteinuria. Thus, enhanced susceptibility to infection in patients with proteinuria is likely caused by mechanisms other than these branches of cellular immune defense.

Given the fact that monocytes are a part of the innate immune response and lymphocytes are part of the adaptive immune response, it is not surprising that elevated circulating monocytes were found in the G-group. In the analysis of the monocyte subsets, the pro-inflammatory subsets were elevated in the G-group, which is generally consistent with earlier findings [24]. However, this could not be confirmed in patients in the P-group. Monocytes of the phenotype Mo1 express different genes coding for innate immune defense against microbial pathogens [25] and are equipped with a high phagocytic capacity [26]. A more inflammatory nature is ascribed to the Mo2 and Mo3 subsets [27]. Mo2 in particular appears to be related to cardiovascular disease [28,29]. Generally, a decreased eGFR and even low-grade proteinuria appear to be associated with arterial stiffness [11]. Therefore, as the expansion of proinflammatory monocytes are linked to the severity of renal impairment [27], we suggest that elevated Mo2 levels are the result of a lower eGFR in our G-group (about 16 mL/min/1.73 m2 lower compared to the P-group).

The adaptive immune system is defined by the interaction of professional antigen-presenting cells, including monocytes, with the corresponding T cells. Mo2 and Mo3 may be able to present antigens to T cells via MHC molecules [25,30]. We therefore measured the expression of the classical MHC class II molecule HLA-DR. However, the expression pattern was not different between the three groups. Effective T-cell activation is also dependent on what are known as co-stimulatory molecules, CD86 (B7-2) on monocytic cells and APCs, and CD28 on T cells. Defective B7-2 signaling in kidney failure patients has been reported in previous studies [9,31]. In our study, we found no differences regarding CD86 expression across the three cohorts. However, it is worth noting that we compared three groups that did not differ regarding their inflammatory and cardiovascular risk profiles.

The question is, therefore, whether there is any indication of a more activated immune system in patients from the P-group. To look more closely at the adaptive immune response in our cohort, we first challenged the PBMCs of the three groups with the rather broad SEB stimulus. This mimics an adaptive immune response by stimulating CD4, CD8, and γ+δ+ T cells by cross-linking the T-cell receptor (outside the peptide binding-groove) with MHC class II molecules (HLA-DR, HLA-DQ, and HLA-DP) on antigen-presenting cells [32,33]. To do so, we stimulated the cells with a very high concentration of SEB to see the “maximal” immune response. Most interestingly, the G-group showed the highest polyclonal immune response in both CD4 T cells and CD8 T cells, indicating the presence of a higher level of activation in these patients.

It has been suggested that uremic toxins play a significant role in T-cell activation and dysfunction, thereby contributing to the immunodeficiency observed in CKD [34,35]. This nonspecific activation could result in T cells exhibiting a reduced capacity to respond to specific antigenic stimulation.

In addition to quantitative changes in circulating lymphocytes, qualitative alterations to T-cell populations have been described in CKD. Hartzell et al. reported an increased frequency of exhausted CD4+ and CD8+ T cells in patients with CKD and ESKD. These cells were characterized by the expression of PD-1 and KLRG1 and impaired cytokine production upon stimulation, suggesting that T-cell exhaustion may contribute to impaired adaptive immune function in CKD [36]. Other studies have reported alterations in the composition of CD4+ T-cell subsets. Lin et al. demonstrated a reduced frequency of activated regulatory T cells, and an increased frequency of Th17 cells in patients with IgA nephropathy, resulting in a reduced Treg/Th17 ratio and indicating a shift towards a more proinflammatory state [37]. Similarly, Gu et al. reported reduced frequencies of regulatory T cells and increased frequencies of Th1 and Th17 cells in patients with type 2 diabetes and macroalbuminuria compared with patients with normo- or microalbuminuria [38]. As a detailed phenotypic characterization of T-cell subsets was not performed in our study, we cannot exclude the possibility that the reduced lymphocyte fractions observed in the P- and G-groups were accompanied by qualitative changes in T-cell composition.

Regarding the effects on antigen-specific immune reactions, the PBMCs of the three groups were challenged with CMV- and SARS-CoV-2-specific antigens. While absolute numbers of cells reacting to nominal antigens were similar in the three patient groups, the relative fraction of cells specifically activated in relation to a polyclonal SEB activation tended to be lower in the G-group. Our cohort was likely too small to prove this effect statistically. This observation thus generates the hypothesis that antigen-specific T-cell activation in individuals with reduced GFR (but not in those with proteinuria) occurs against a background of high nonspecific cellular inflammation.

CXCL10 mRNA expression is regarded as a proxy for SARS-CoV-2-specific immune responses [18,39]. Using this technique, we can confirm SARS-CoV-2 antigenic immune responses as measured by flow cytometry. Although we think our data are consistent, CXCL10 fulfills multiple functions [19]; hence, its significance should be re-evaluated by studying a larger cohort of patients with reduced eGFR and severe albuminuria.

### Study Limitations

Our study has some limitations, among them the small number of patients in each group. Antigen-specific activation has a high interindividual variability that hinders experimental measurement. Furthermore, we cannot be sure that elevated polyclonal T-cell activation is a consequence of altered T-cell function; the high numbers of proinflammatory monocytes in the circulation might be an alternative explanation.

## 4. Materials and Methods

### 4.1. Study Population

The cross-sectional study included 30 control patients (C) (eGFR > 60 mL/min/1.73 m^2^ and uACR < 300 mg/g), 19 patients with an eGFR < 60 mg/mL/1.73 m^2^ and a uACR < 300 mg/g (G-group), and 25 patients with a uACR > 300 mg/g (P-group) independent of their eGFR.

Exclusion criteria were age < 18 or >89 years; active use of renal replacement therapy; use of any kind of immunosuppressive therapy within the preceding 4 weeks or treatment with rituximab, cyclophosphamide, or obinutuzumab within 12 months prior to enrollment; clinical/paraclinical signs of infection within 2 weeks prior to enrollment; Child–Pugh class C liver cirrhosis; active malignancy; blood transfusion within 4 weeks prior to enrollment; and pregnancy.

There were no significant differences in the patients’ cardiovascular medical histories across the three cohorts. All patients were routinely checked at the outpatient and inpatient departments of Internal Medicine Clinic II of the University of Halle-Wittenberg. The study was conducted according to the Declaration of Helsinki. Written informed consent was obtained from all study subjects, and the study protocol was approved by the local ethics committee. The protocol is listed with the German Clinical Trial Register (DRKS00035382). Blood samples were drawn in the morning during regular visits.

### 4.2. Absolute Cell Count Determination

Trucount tubes (BD Biosciences, Heidelberg, Germany) were used to determine absolute cell count numbers in 50 µL of whole blood. Total leukocytes were identified by anti-CD45 (Miltenyi Biotec, Bergisch-Gladbach, Germany); granulocytes by anti-CD15 (Thermo Fisher Scientific, Darmstadt, Germany); monocytes by anti-CD86, HLA-DR (both Thermo Fisher Scientific), anti-CD14, and anti-CD16 (both Miltenyi Biotec); and lymphocytes by anti-CD3 (Miltenyi Biotec) and HLA-DR (Thermo Fisher Scientific). Monocyte subsets were gated according to Girndt et al.

### 4.3. PBMC Isolation

PBMCs were isolated by Ficoll density centrifugation (GE Healthcare, Solingen, Germany) from EDTA blood samples drawn during each patient’s regular visit. The quality of isolated cells was tested by 7-AAD staining (Thermo Fisher Scientific). The vitality of PBMC was 99.4% ± 0.6 for CO, 99.2 ± 0.8 for CKD, and 99.5 ± 0.3 for P.

### 4.4. Stimulation of PBMCs with CMV, SARS-CoV-2, SEB, and Intracellular CD137 Staining

Aliquots of 1.0 × 10^6^ cells each were incubated in 500 µL RPMI 1640 medium (Sigma-Aldrich, St. Louis, MO, USA) containing 1% glutamine or 1% penicillin/streptomycin and 10% fetal bovine serum (Thermo Fisher Scientific). All samples were stimulated with a mixture of CD28 (100 µg/mL, Miltenyi Biotec) and CD49d (1 mg/mL, Thermo Fisher Scientific); the supplementation of a low concentration of CD28 was used to detect different immune responses in patients with impaired CD28 expression. Subsequently, the cells were either left untreated (Co) or stimulated with PepTivator CMV pp65 / CMV IE1 (1 µg/mL, Miltenyi Biotec), PepTivator SARS-CoV-2 (Prot_S complete, 1 µg/mL, Miltenyi Biotec), or SEB (1 mg/mL, Sigma-Aldrich). After a short initial incubation period of 30 min at 37 °C in a 5% CO2 atmosphere, 0.5 µL of monensin (1000x, Biolegend, Koblenz, Germany) was added to all samples, followed by a second incubation period over 20 h at 37 °C in a 5% CO_2_ atmosphere. After washing with PBS, THE cells were stained with Viobility Dye 405 (Miltenyi Biotec), followed by fixation (1%PFA, Carl-Roth, Karlsruhe, Germany) and saponification (0,1% saponin, Sigma-Aldrich). Intracellular staining was performed in the presence of saponin using the following antibodies: anti-CD3, -CD8, -CD69 (all Miltenyi Biotec), -CD4 (Thermo Fisher Scientific), and -CD137 (Biolegend). CD4+CD69+CD137+ and CD8+CD69+CD137+ cells were determined using a MAQS Quant flow cytometer and the MACS Quantify software package (Miltenyi Biotec; Software version: 2.8.1618.16380). All samples were adjusted to FMO controls (fluorescence minus one). For the analysis of T-cell activation, the percentage of activated cells in the corresponding unstimulated sample was subtracted from the percentage measured after stimulation. Therefore, the reported values represent the stimulation-induced change in T-cell activation.

### 4.5. RNA/cDNA/qPCR

RNA was isolated from PBMC lysates using the Direct-zol MiniPrep Plus Isolation Kit (ZymoResearch, Freiburg, Germany). The RNA concentration and the quality of the different fractions were tested using the Z-Tecan Infine^®^200 Pro technique (Tecan Group, Männedorf, Germany).

Equal amounts of RNA (100 ng) were reverse transcribed using the High-Capacity cDNA Reverse Transcription Kit (Thermo Fisher Scientific, Darmstadt, Germany).

The mRNA expressions of CXCL10 (Hs00171042_m1), IFN-γ (Hs00989291_m1), IFN-α (Hs03044218_g1), CD28 (Hs01007422_m1), and RPL37A (Hs01102345_m1) were analyzed using TaqMan probes (Thermo Fisher Scientific) and the qPCRBIO Probe Mix High-ROX (Nippon Genetics, Düren, Germany). The samples were processed in duplicate on a StepOnePlus Cycler (Thermo Fisher Scientific). Data were normalized to RPL37A and expressed relative to the mRNA of a healthy subject (using the ddCt method).

### 4.6. ELISAs

IFN-α and IFN-γ were measured using Human IFN-α and IFN-γ ELISA Max™ Deluxe Sets (Biolegend). The cytomegalovirus IgG ELISA was used to detect IgG antibodies to CMV in human serum (Antikörper-online.de, Aachen, Germany).

### 4.7. Statistics

Results are expressed as mean ± SD. All continuous variables were controlled for normal distribution using the D’Agostino–Pearson omnibus test. Continuous data were compared by one-way ANOVA followed by Tukey’s post hoc test or the Kruskal–Wallis test as appropriate. Categorical variables were analyzed by chi-square test. All calculations were carried out using the SPSS 21.0 (SPSS Inc., Chicago, IL, USA) or GraphPad Prism 9.2.0 statistics software packages (GraphPad Software Inc., La Jolla, CA, USA). The level of significance was set at *p* < 0.05.

## 5. Conclusions

While patients with reduced eGFR (CKD G3-5) show abnormal monocyte subpopulation numbers and T-cell activation, patients with severe proteinuria (CKD A3) do not. Therefore, alternative explanations for the high clinical susceptibility to infection in proteinuric patients need to be discussed.

## Figures and Tables

**Table 1:** Demographic data of the patient groups with proteinuria (P), reduced GFR (G), or normal kidney function (C). Data are presented as mean ± standard deviation unless otherwise stated. Comparisons were performed by ANOVA and Dunn’s post hoc test or chi-square test as appropriate.

	P (n = 25)	G (n = 19)	C (n = 30)	*p*
Age (years)	52.9 ± 18.2	65.7 ± 11.9	57.7 ± 19.3	0.0578
Gender (%, f)	40.0	47.3	66.7	0.124
BMI (kg/m^2^)	28.5 ± 6.8	28.9 ± 7.5	28.4 ± 6.1	0.962
Diabetes (%)	62.5	38.9	34.5	0.104
eGFR (mL/min/1.73 m^2^) [Median: Min-Max]	41.2 ± 29.0	25.4 ± 10.2	86.0 ± 24.7	<0.0001
				P vs. C ****
				G vs. C ****
	[37.7: 7.48–107.2]	[27.4: 12.7–42.3]	[80.2: 45.9–135.6]	
Creatinine (µmol/L)	233.8 ± 177.2	232.9 ± 128.6	79.1 ± 18.5	<0.0001
				P vs. C ****
				G vs. C ***
Cystatin C (mg/L)	2.4 ± 1.3	2.6 ± 0.7	1.0 ± 0.3	<0.0001
				P vs. C ****
				G vs. C ****
CRP (mg/L)	6.7 ± 13.4	6.9 ± 13.7	5.5 ± 10.7	0.903
IL-6 (pg/mL)	6.6 ± 6.3	8.7 ± 9.1	4.7 ± 4.4	0.121
Albumine (g/L)	38.5 ± 8.3	41.2 ± 5.4	41.8 ± 3.5	0.120
uACR (mg/g)	1692.0 ± 1487.0	28.0 ± 27.6	18.2 ± 26.4	<0.0001
				P vs. G ****
[Median: Min-Max]	[990.1: 300.1–4400.0]	[13.0: 2.0–90.3]	[7.4: 3.7–107.7]	P vs. C ****
Hemoglobin (mmol/L)	7.8 ± 1.7	7.7 ± 1.7	8.0 ± 1.1	0.738
CAD (%)	24.0	26.3	20.0	0.867
CVD (%)	20	15.8	10.0	0.579
Hypertension (%)	87.5	78.9	78.6	0.664
Angiotensin blockers (%)	84.0	94.7	70.0	0.089
SGLT2-inhibitor (%)	48.0	47.4	6.9	0.0012
Diuretics (%)	40.0	57.9	27.6	0.111

*** *p* < 0.001, **** *p* < 0.0001.

**Table 2:** Absolute leukocyte numbers and the relative contributions of different cell types in the hematology analysis. Data are presented as mean ± standard deviation. Comparisons were performed by ANOVA and Dunn’s post hoc test.

	P (N = 25)	G (N = 19)	C (N = 30)	*p*
Leucocytes (×10^6^/mL)	7.4 ± 2.6	6.8 ± 1.5	6.9 ± 1.8	0.563
Granulocytes (% of Leuco)	65.0 ± 9.9	63.5 ± 7.6	57.5 ± 10.0	0.013
				P vs. C *
Lymphocytes (% of Leuco)	23.9 ± 8.5	23.2 ± 6.9	30.8 ± 9.3	0.004
				P vs. C *
				G vs. C *
Monocytes (% of Leuco)	7.2 ± 2.3	9.2 ± 2.3	8.1 ± 2.0	0.016
				P vs. G *
Eosinophiles (% of Leuco)	3.0 ± 1.7	2.9 ± 2.8	2.6 ± 1.3	0.706

* *p* < 0.05.

**Table 3:** Cellular response to CMV- and SARS-CoV-2-specific stimulation in the control (C), G-, and P-groups, as well as in the subgroup of CMV-seropositive individuals (Sero+). Values are presented as mean ± standard deviation (SD) and analyzed using one-way ANOVA or the Kruskal–Wallis test as appropriate.

	P	G	C	*p*
CMV-CD4+69+137+ (%)	0.56 ± 0.64	0.30 ± 0.32	0.46 ± 0.48	0.50
CMV-CD4+69+137+ Sero+ (%)	0.88 ± 0.77	0.58 ± 0.26	0.85 ± 0.63	0.65
CMV-CD8+69+137+ (%)	1.89 ± 2.97	1.48 ± 2.35	1.60 ± 4.5	0.27
CMV-CD8+69+137+ Sero+ (%)	2.98 ± 4.12	3.77 ± 2.97	4.78 ± 8.89	0.48
SARS-CoV-2-CD4+69+137+ (%)	0.45 ± 0.62	0.21 ± 0.24	0.21 ± 0.22	0.58
SARS-CoV-2-CD8+69+137+ (%)	0.23 ± 0.55	0.14 ± 0.24	0.19 ± 0.27	0.33

**Table 4:** Interferon levels in the serum of patients.

	P	G	C	*p*
IFN-γ (pg/mL) Mean [Min.; Max.]	0.12 [0.04; 0.76]	0.08 [0.07; 0.1]	0.09 [0.03; 0.2]	0.966
IFN-α (pg/mL) Mean [Min.; Max.]	7.1 [0; 109]	13.9 [0; 162.3]	6.12 [0; 31.8]	0.505

Data are presented as means [Min.; Max.].

**Table 5:** Correlation of CXCL10, IFN- γ mRNA expression, and CMV- and SARS-CoV-2-specific immune responses.

	CXCL10 mRNA Expression	IFN-γ mRNA Expression
%CMV-CD4+69+137+	r = 0.003	r = 0.252
	*p* = 0.984	*p* = 0.043 *
%CMV-CD8+69+137+	r = 0.050	r = 0.266
	*p* = 0.691	*p* = 0.032 *
%SARS-CoV2-CD4+69+137+	r = −0.130	r = −0.226
	*p* = 0.304	*p* = 0.070
%SARS-CoV2-CD8+69+137+	r = −0.200	r = −0.011
	*p* = 0.110	*p* = 0.926

Data are presented as mean ± SD. * *p* < 0.05.

## Data Availability

The original contributions presented in this study are included in the article. Further inquiries can be directed to the corresponding author.

## Abbreviations

The following abbreviations are used in this manuscript:

ACE	angiotensin converting enzyme
ARB	angiotensin receptor blocker
CAD	coronary artery disease
CAVD	coronary arterio-venous disease
CKD	chronic kidney disease
CMV	cytomegalovirus
C-group	control group
P-group	patients with severe albuminuria (uACR > 300 mg/g)
G-group	patients with eGFR < 60 mg/mL/1.73 m^2^ and uACR < 300 mg/g
CRP	C-reactive protein
CXCL10	C-X-C motif chemokine ligand 10
eGFR	estimated glomerular filtration rate
IFN	interferon
SARS-CoV-2	severe acute respiratory syndrome coronavirus 2
SEB	Staphylococcus enterotoxin B
SGLT2	sodium glucose co-transporter-2
uACR	urinary albumin-to-creatin ratio
